# The rise in preanalytical errors during COVID-19 pandemic

**DOI:** 10.11613/BM.2021.020710

**Published:** 2021-06-15

**Authors:** Tapasyapreeti Mukhopadhyay, Arulselvi Subramanian, Shivam Pandey, Nirupam Madaan, Anjan Trikha, Rajesh Malhotra

**Affiliations:** 1 Department of Laboratory Medicine, Jai Prakash Narayan Apex Trauma Centre, All India Institute Medical Sciences, New Delhi, India; 2 Department of Biostatistics, All India Institute Medical Sciences, New Delhi, India; 3 Hospital Administration, Jai Prakash Narayan Apex Trauma Centre, All India Institute Medical Sciences, New Delhi, India; 4 Department of Anaesthesis and Critical care, Jai Prakash Narayan Apex Trauma Centre, All India Institute Medical Sciences, New Delhi, India; 5 Department of Orthopedics, Jai Prakash Narayan Apex Trauma Centre, All India Institute Medical Sciences, New Delhi, India

**Keywords:** laboratory biosafety, preanalytical phase, quality indicators, SARS-CoV-2, total testing process

## Abstract

**Introduction:**

The COVID-19 pandemic has posed several challenges to clinical laboratories across the globe. Amidst the outbreak, errors occurring in the preanalytical phase of sample collection, transport and processing, can further lead to undesirable clinical consequences. Thus, this study was designed with the following objectives: (i) to determine and compare the blood specimen rejection rate of a clinical laboratory and (ii) to characterise and compare the types of preanalytical errors between the pre-pandemic and the pandemic phases.

**Materials and methods:**

This retrospective study was carried out in a trauma-care hospital, presently converted to COVID-19 care centre. Data was collected from (i) pre-pandemic phase: 1^st^ October 2019 to 23^rd^ March 2020 and (ii) pandemic phase: 24^th^ March to 31^st^ October 2020. Blood specimen rejection rate was calculated as the proportion of blood collection tubes with preanalytical errors out of the total number received, expressed as percentage.

**Results:**

Total of 107,716 blood specimens were screened of which 43,396 (40.3%) were received during the pandemic. The blood specimen rejection rate during the pandemic was significantly higher than the pre-pandemic phase (3.0% versus 1.1%; P < 0.001). Clotted samples were the commonest source of preanalytical errors in both phases. There was a significant increase in the improperly labelled samples (P < 0.001) and samples with insufficient volume (P < 0.001), whereas, a significant decline in samples with inadequate sample-anticoagulant ratio and haemolysed samples (P < 0.001).

**Conclusion:**

In the ongoing pandemic, preanalytical errors and resultant blood specimen rejection rate in the clinical laboratory have significantly increased due to changed logistics. The study highlights the need for corrective steps at various levels to reduce preanalytical errors in order to optimise patient care and resource utilisation.

## Introduction

Ever since the pandemic was declared on 11^th^ March, 2020 by the World Health Organisation, it has posed several challenges on various fronts. The clinical laboratories across the globe have adapted to maintain the highest standards of patient care despite the COVID-19 pandemic. The imprecision and inaccuracies in results occurring due to preanalytical errors have always been a major cause of concern for diagnostic laboratories (*1*-*4*). Preanalytical errors range between 46‐68% out of all errors occurring in the total testing process (*5*). The preanalytical errors result in specimen rejection due to which another blood specimen has to be collected and sent again to the laboratory. This results in a significant delay in receiving laboratory test results which often compromises patient care, especially for patients in intensive care units. The cost burden due to preanalytical errors has been reported to constitute 0.2-1.2% of the total hospital operating costs (*6*).

During the ongoing pandemic, the healthcare professionals are required to wear personal protective equipment (PPE) while taking care of the patients. Furthermore, the specimen collection and transport logistics are also different from the pre-pandemic times (*7*, *8*). The impact of these changed protocols for specimen collection, packaging and transport on the frequency of preanalytical errors is unknown. We hypothesised that during this ongoing pandemic, the rate and characteristics of preanalytical errors is likely to differ from the pre-pandemic times. Basic knowledge of these differences can help to focus on strategies to prevent preanalytical errors during future pandemics and in similar exigencies. Therefore, we designed this study with the following objectives: (i) to determine and to compare the blood specimen rejection rate of our clinical laboratory between the pre-pandemic and the pandemic phases, and (ii) to characterise and to compare the type of preanalytical errors between the pre-pandemic and the pandemic phases.

## Materials and methods

### Materials

This retrospective observational study was conducted in the clinical laboratory of a tertiary care hospital caring for trauma patients in South Asia. The centre was declared as a COVID-19 care centre on 23^rd^ March 2020 to take care of the anticipated rise in the cases. The central laboratory of this 250 bedded hospital receives blood samples for complete blood count, coagulation profile, biochemical examination, immunology, along with samples for urinalysis and histopathology and operates 24x7 throughout the year. The laboratory process is monitored daily by internal quality controls. The phlebotomies of the hospitalised patients are performed either by treating physicians or nurses.

For the study, data was retrospectively collected for the period from October 2019 to October 2020. The study period was divided into: (i) the pre-pandemic phase from 1^st^ October 2019 to 23^rd^ March 2020 and (ii) the pandemic phase from 24^th^ March to 31^st^ October 2020.

All specimens received in the clinical laboratory during the study period were included. Laboratory records for the specimens rejected due to preanalytical errors were accessed for the study period and analysed. The sample rejection rate was calculated as the proportion of blood samples with preanalytical errors out of the total number of blood specimens received and was expressed as a percentage.

### Methods

The vacutainer blood collection tubes (BCT) with potassium salt of ethylenediaminetetraacetic acid (EDTA) and sodium citrate are used for complete blood count and coagulation profile, respectively. The serum separator tube with silica clot activator, polymer gel, silicone-coated interior (Becton Dickinson, Franklin Lakes, USA) are used for biochemical and immunological tests. During the current pandemic, the specimens collected from patients in these barcoded blood collection tubes, are being manually delivered to the laboratory by a hospital attendant in contrast to the pneumatic shoots which were used in the pre-pandemic phase.

Currently, all specimens received in the clinical laboratory are processed in class 2 biological safety cabinet by a medical technologist after wearing PPE. The BCT are at first treated with ultraviolet rays for five minutes, after which the outer surface of the BCT are wiped with 0.1% hypochlorite solution using tissue paper and kept for five minutes before further processing. All the BCT received in the laboratory are visually screened by the medical technologist for the following seven quality indicators (QI): QI-1 - number of improperly/mislabelled BCT, QI-2 - number of inappropriate BCT (wrong BCT for the requested test), QI-3 - number of BCT with insufficient volume, QI-4 - number of BCT with inadequate sample-anticoagulant ratio, QI-5 - number of clotted samples, QI-6 - number of haemolysed samples and QI-7 - number of samples with lipaemia (elaborated in Supplementary Table 1) at the sample receiving area. Colour of the serum or plasma obtained after centrifugation is visually inspected; pink or red colour is suggestive of haemolysis and turbid or white/creamy colour is suggestive of lipaemia. The BCT with any of the above mentioned preanalytical error(s) is rejected after informing the healthcare professional at the source site, and the information is recorded systematically. The samples with no errors are processed and processing is followed by the technical validation of the generated results.

Also, during the study period, a new laboratory information system (LIS) was introduced in July (e-Hospital, developed by National Informatics Centre under the Digital India initiative of the Ministry of Electronics & Information Technology, Government of India) in the clinical laboratory due to the termination of the hospital contract of the platform being used earlier. In order to get acquainted with the user experience and design of the new platform, the laboratory staff and doctors underwent three days training.

### Statistical analysis

Data collected is descriptive and is represented in percentages. Data was analysed using Stata Statistical Software, version 15 (StataCorp 2017, College Station, USA). Categorical variables were described using frequencies and percentages and the two groups were compared using the chi-square test. A value of P < 0.05 was considered statistically significant.

## Results

**Figure 1:**
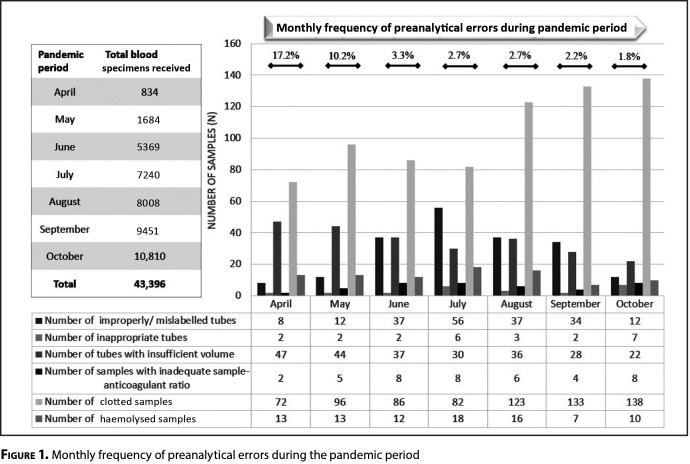
Monthly frequency of preanalytical errors during the pandemic period

Total of 107,716 blood specimens were screened of which 43,396 (40.3%) were received during the pandemic phase (Table 1). The blood specimen rejection rate during the pandemic phase was significantly higher than the pre-pandemic phase (3.0% *versus* 1.1%; P < 0.001). On monthly analysis, most errors in the pandemic phase occurred in the initial months, highest in April (17.2%) followed by May (10.2%) and least in October (1.8%) (Figure 1).

**Table 1:** Blood specimen rejection rate and characterisation of preanalytical errors in the pre-pandemic and the pandemic phases

**Variables**	**Pre-pandemic phase** **(N = 64,320)**	**Pandemic phase** **(N = 43,396)**	**P**
**Blood specimen rejection rate**	1.1%	3.0%	< 0.001
**Total BCT rejected**	735	1324	< 0.001
**Proportion of BCT with individual preanalytical errors out of the total number received**			
**Improperly/mislabelled BCT**	32/703	196/1128	< 0.001
**Inappropriate BCT**	10/725	24/1300	0.300
**BCT with insufficient volume**	22/713	244/1080	< 0.001
**Inadequate sample-anticoagulant ratio**	47/688	41/1283	0.003
**Clotted samples**	166/569	594/730	< 0.001
**Haemolysed samples**	215/520	89/1235	< 0.001
BCT - blood collection tube. Level of significance set at P < 0.05.			

### Characterisation of preanalytical errors

Most of the preanalytical errors in the pandemic phase were due to undesirable clotting of the samples (730/1324; 55.1%) which remained high during the entire period. Moreover, there was a significant increase in improperly labelled samples (P < 0.001), highest observed in the month of July. Samples with insufficient volume also increased significantly during the pandemic period (P < 0.001). A significant decline in the samples with inadequate sample-anticoagulant ratio (P = 0.003) and haemolysed samples (P < 0.001) were observed (Table 1).

## Discussion

The major finding of our study was that the specimen rejection rate during the pandemic phase was significantly higher than the pre-pandemic phase which was highest at the beginning of the pandemic and gradually declined towards the end of the study period. In contrast, the other published studies which compare the preanalytical errors occurring in the pre-pandemic and pandemic phase, have not shown any change in the frequency of preanalytical errors during the pandemic period in their set-up (*9*, *10*). However, the overall rejection rate observed in our study was similar to pre-pandemic studies (*11*-*15*).

The overall increased frequency of preanalytical errors is probably due to three reasons. Firstly, there was increase in the number of healthcare professionals handling patient specimens *per* day in the wards and intensive care units. This was due to shorter duration of duty shifts and frequent biweekly/monthly rotation of healthcare professionals due to mandatory quarantine periods. Also the healthcare professionals involved in phlebotomy were from a mix of clinical and non-clinical specialties. The increased number and heterogeneity of healthcare professionals working in a day may have affected the quality of sampling. Secondly, the PPE worn by all healthcare personnel made specimen collection challenging. The decreased field of vision, reduced manual dexterity along with the heightened mental stress experienced while managing infectious patients are possible reasons for increase in some of the errors in the preanalytical phase (*16*-*18*). Lastly, the shortage of time for the healthcare professionals due to high patient load may also have affected specimen collection process. Appointing a trained and dedicated team of phlebotomists may help to reduce preanalytical errors and relieve the burden on healthcare professionals involved in clinical care of the patients.

The sharp increase in the frequency of preanalytical errors in the initial months of the pandemic followed by its rapid decline, could be explained by the fact that a basic level of competency was achieved by the healthcare professionals as the pandemic continued, along with the decrease in fear and anxiety of the healthcare professionals while collecting and handling blood specimens.

In our study, clotted samples (QI-5) constituted the largest proportion of preanalytical errors both in the pre-pandemic and the pandemic phases, and continued to remain significantly high during the entire pandemic period. Clotting of blood in BCT with anticoagulants generally occurs either due to improper mixing after blood collection or due to improper blood-to-anticoagulant ratio. The under-filled or over-filled citrated tubes with inappropriate sample-anticoagulant ratio (QI-4) that may or may not have visible clots can affect test results and hence be detrimental to patient care (*19*). This study finding can be attributed probably to varying level of knowledge and awareness amongst the healthcare professionals regarding good phlebotomy practices, and the number of inversions recommended to mix anticoagulant and blood properly. The added stress of focussing on maintaining stringent infection control measures could also have resulted in increase in this error.

*In-vitro* haemolysis (QI-6) is usually reported to be the commonest cause of preanalytical error in a clinical chemistry laboratory. In our study, it was the second most common error in the pre-pandemic period and the fourth most common error during the pandemic period. Undesirable haemolysis may occur either due to high pressure of the piston during collecting and dispensing the blood in tubes or could be due to exposure to extreme temperature or vigorous shaking during transport (*20*). In the pre-pandemic phase, the blood samples were received mostly by pneumatic shoot, and rarely hand-delivered by a hospital attendant. Also, due to the urgency at the clinician’s end to fetch reports as quickly as possible, the hospital attendant transporting the specimens hurried to the laboratory, probably deviating from the specimen handling protocol during transport. However, at present, extra measures and safety protocols were implemented while handling blood specimens of COVID-19 patients. The specimens are transported to the laboratory in bio-hazard zip-lock bags placed inside dedicated blood transport box. This change of practice may have led to reduction in the frequency of haemolysed samples. Standardising the specimen transport protocol and its strict execution can probably help in reducing this error even in the non-pandemic times in the future (*21*).

Samples with insufficient volumes (QI-3) formed a major proportion during the pandemic period and were significantly higher than the pre-pandemic phase. Generally, the major causes for receiving insufficient quantity for testing are difficult access for venepuncture in patients with chronic debilitating diseases, patients on chemotherapy, patients in shock and in the paediatric population. The use of microtainers and micro-tubes to reduce the volume withdrawn for such patients may aid in reducing this error. Apart from the above reasons, increased demand of serum based test parameters like procalcitonin, interleukin-6, ferritin and troponin-I could have resulted in increased samples with insufficient volume. Another reason could be that in the pre-pandemic period, the medical technologist used to de-cap the BCT to collect the serum in a separate cup and run it on manual mode in order to decrease the dead space in case of low sample volume. This was done to prevent repeat sampling and thereby avoid losing time during management of critically ill trauma patients. But presently, de-capping any BCT is not recommended in the laboratory, considering the risk of infection to the laboratory personnel.

The introduction of a new LIS, a new barcode generating system along with addition of new test profile during the second half of the pandemic phase (July 2020) in the hospital led to a significant increase in mislabelled samples (QI-1). The healthcare professionals at patient care sites took time to learn and use the new LIS, the new barcode labels and their generation method. Some of the common errors observed during the initial introduction of the new LIS were generation of wrong bar codes in terms of wrong test selection, wrong patient selection or re-selection of an old barcode. This finding is a confounding factor in our study and could have been avoided if the same LIS had been in use throughout the study period.

The errors due to mislabelling of tubes affect patient identification, and may lead to patient identity mix-up. Recommendations for the correct patient and sample identification procedures should be strictly followed at the patient site and in the clinical laboratory (*22*).

The frequency of blood samples received in inappropriate BCT (QI-2) during both the phases were similar and reflects a general lack of knowledge among the healthcare professionals on the rationale of using anti-coagulated and plain/serum-separator BCT (*23*).

It is evident from our study that during the pandemic, preanalytical errors have increased due to the changed logistics and therefore require more attention (Supplementary Table 2). Most can be addressed by training the healthcare professionals and by periodic reiteration by experts about the sample withdrawal technique, the order of draw, the number of inversions recommended to mix anticoagulant and blood properly, and the required standardised volume to be dispensed for each blood collection tube based on the available international guidelines (*24*-*26*). A special teaching module on ‘laboratory preparedness during a pandemic with a focus on preventing preanalytical errors’ for healthcare professionals working outside and inside the laboratory should be designed and implemented. Better communication between laboratories and the healthcare professionals at patient care sites can definitely help in overcoming the concerning issue of preanalytical errors.

There were certain limitations to our study. The frequency and the distribution of preanalytical errors observed in this study may vary at different centres. Since specimens were visually screened for preanalytical QIs, mild haemolysis or micro-clots may may not have been recorded that may potentially change the proportion of errors. The introduction of a new LIS during the study period led to a significant increase in the mislabelled samples and is a confounding factor.

## Conclusion

Although the number of specimens received during the pandemic period has declined, the frequency of preanalytical errors and the resultant blood specimen rejection rate has risen significantly in comparison to the pre-pandemic time. Our study highlights the need for corrective steps at various levels to reduce preanalytical errors which shall help to optimise patient care and resource utilisation.

## References

[1] Lippi G Blanckaert N Bonini P Green S Kitchen S Palicka V Causes, consequences, detection, and prevention of identification errors in laboratory diagnostics. Clin Chem Lab Med. 2009;47:143–53. 10.1515/CCLM.2009.045 19099525

[2] Bonini P Plebani M Ceriotti F Rubboli F. Errors in laboratory medicine. Clin Chem. 2002;48:691–8. 10.1093/clinchem/48.5.691 11978595

[3] Lippi G. Governance of preanalytical variability: Travelling the right path to the bright side of the moon? Clin Chim Acta. 2009;404:32–6. 10.1016/j.cca.2009.03.026 19302993

[4] Cornes M. The preanalytical phase - Past, present and future. Ann Clin Biochem. 2020;57:4–6. 10.1177/0004563219867989 31324119

[5] Green SF. The cost of poor blood specimen quality and errors in preanalytical processes. Clin Biochem. 2013;46:1175–9. 10.1016/j.clinbiochem.2013.06.001 23769816

[6] Plebani M. Quality indicators to detect pre-analytical errors in laboratory testing. Clin Biochem Rev. 2012;33:85–8. 22930602 PMC3428256

[7] Loh TP Horvath AR Wang CB Koch D Lippi G Mancini N International Federation of Clinical Chemistry and Laboratory Medicine Taskforce on COVID-19. Laboratory practices to mitigate biohazard risks during the COVID-19 outbreak: an IFCC global survey. Clin Chem Lab Med. 2020;58:1433–40. 10.1515/cclm-2020-0711 32549123

[8] Loh TP Horvath AR Wang CB Koch D Adeli K Mancini N International Federation of Clinical Chemistry and Laboratory Medicine Taskforce on COVID-19. Operational considerations and challenges of biochemistry laboratories during the COVID-19 outbreak: an IFCC global survey. Clin Chem Lab Med. 2020;58:1441–9. 10.1515/cclm-2020-0710 32549122

[9] Frater JL Anderson J. The impact of biosafety enhancement on stat laboratory quality metrics: Lessons from the COVID-19 pandemic. Clin Chim Acta. 2021;512:58–62. 10.1016/j.cca.2020.11.021 33285119 PMC7836754

[10] Ongen-Ipek B Sitar ME Karadeniz A. Adaptation of Clinical Laboratories to COVID 19 Pandemic: Changes in Test Panels, Overcoming Problems and Preparation Suggestions for Future Pandemics Adaptation of Clinical Laboratories to COVID 19 Pandemic. Clin Lab. 2020;66: 10.7754/Clin.Lab.2020.200624 33180431

[11] Sakyi A Laing E Ephraim R Asibey O Sadique O. Evaluation of analytical errors in a clinical chemistry laboratory: a 3 year experience. Ann Med Health Sci Res. 2015;5:8–12. 10.4103/2141-9248.149763 25745569 PMC4350069

[12] Sonmez C Yıldız U Akkaya N Taneli F. Preanalytical Phase Errors: Experience of a Central Laboratory. Cureus. 2020 March;12:e7335. 10.7759/cureus.7335 32313776 PMC7164707

[13] Tapper MA Pethick JC Dilworth LL McGrowder DA. Pre-analytical errors at the chemical pathology laboratory of a teaching hospital. J Clin Diagn Res. 2017;11:BC16–8. 10.7860/JCDR/2017/30159.10378 28969112 PMC5620752

[14] Chawla R Goswami B Tayal D Mallika V. Identification of the types of preanalytical errors in the clinical chemistry laboratory: 1-year study at GB Pant Hospital. Lab Med. 2010;41:89–92. 10.1309/LM9JXZBMLSVJT9RK

[15] Arul P Pushparaj M Pandian K Chennimalai L Rajendran K Selvaraj E Prevalence and types of preanalytical error in hematology laboratory of a tertiary care hospital in South India. J Lab Physicians. 2018;10:237–40. 10.4103/JLP.JLP_98_17 29692594 PMC5896195

[16] Frater JL Anderson J. The impact of biosafety enhancement on stat laboratory quality metrics: Lessons from the COVID-19 pandemic. Clin Chim Acta. 2021;512:58–62. 10.1016/j.cca.2020.11.021 33285119 PMC7836754

[17] Ongen-Ipek B Sitar ME Karadeniz A. Adaptation of Clinical Laboratories to COVID 19 Pandemic: Changes in Test Panels, Overcoming Problems and Preparation Suggestions for Future Pandemics Adaptation of Clinical Laboratories to COVID 19 Pandemic. Clin Lab. 2020;66: 10.7754/Clin.Lab.2020.200624 33180431

[18] Daugherty EL Perl TM Needham DM Rubinson L Bilderback A Rand CS. The use of personal protective equipment for control of influenza among critical care clinicians: A survey study. Crit Care Med. 2009;37:1210–6. 10.1097/CCM.0b013e31819d67b5 19242326

[19] Forgie SE Reitsma J Spady D Wright B Stobart K. The “fear factor” for surgical masks and face shields, as perceived by children and their parents. Pediatrics. 2009;124:e777–81. 10.1542/peds.2008-3709 19786438

[20] Tan BY Chew NW Lee GK Jing M Goh Y Yeo LL Psychological impact of the COVID-19 pandemic on health care workers in Singapore. Ann Intern Med. 2020;173:317–320. 10.7326/M20-1083 32251513 PMC7143149

[21] Lippi G Salvagno GL Montagnana M Lima-Oliveira G Guidi GC Favaloro EJ. Quality standards for sample collection in coagulation testing. Semin Thromb Hemost. 2012;38:565–75. 10.1055/s-0032-1315961 22669757

[22] Clinical Laboratory Standards Institute (CLSI). Collection, Transport, and Processing of Blood Specimens for Testing Plasma-Based Coagulation Assays and Molecular Hemostasis Assays. CLSI H21-A5 document. Wayne, PA: CLSI; 2008.

[23] Clinical Laboratory Standards Institute (CLSI). Procedures for the handling and processing of blood specimens for common laboratory tests. CLSI H18-A4 document. Wayne, PA: CLSI; 2010.

[24] van Dongen-Lases EC Cornes MP Grankvist K Ibarz M Kristensen GB Lippi G Working Group for Preanalytical Phase (WG-PRE), European Federation of Clinical Chemistry and Laboratory Medicine (EFLM). Patient identification and tube labelling - a call for harmonisation. Clin Chem Lab Med. 2016;54:1141–5. 10.1515/cclm-2015-1089 26816400

[25] Bayot ML, Tadi P. Laboratory Tube Collection. [Updated 2020 Nov 17]. In: StatPearls. Available at: https://www.ncbi.nlm.nih.gov/books/NBK555991/InStatPearls 2020 Mar 5. Accessed January 20th 2021

[26] Narayanan S. The preanalytic phase: an important component of laboratory medicine. Am J Clin Pathol. 2000;113:429–52. 10.1309/C0NM-Q7R0-LL2E-B3UY 10705825

